# A Robust Tracking-by-Detection Algorithm Using Adaptive Accumulated Frame Differencing and Corner Features

**DOI:** 10.3390/jimaging6040025

**Published:** 2020-04-21

**Authors:** Nahlah Algethami, Sam Redfern

**Affiliations:** School of Computer Science, National University of Ireland Galway, University Road, H91 TK33 T Galway, Ireland; sam.redfern@nuigalway.ie

**Keywords:** track-by-detection, motion features, overhead camera, smart meeting

## Abstract

We propose a tracking-by-detection algorithm to track the movements of meeting participants from an overhead camera. An advantage of using overhead cameras is that all objects can typically be seen clearly, with little occlusion; however, detecting people from a wide-angle overhead view also poses challenges such as people’s appearance significantly changing due to their position in the wide-angle image, and generally from a lack of strong image features. Our experimental datasets do not include empty meeting rooms, and this means that standard motion based detection techniques (e.g., background subtraction or consecutive frame differencing) struggle since there is no prior knowledge for a background model. Additionally, standard techniques may perform poorly when there is a wide range of movement behaviours (e.g. periods of no movement and periods of fast movement), as is often the case in meetings. Our algorithm uses a novel coarse-to-fine detection and tracking approach, combining motion detection using adaptive accumulated frame differencing (AAFD) with Shi-Tomasi corner detection. We present quantitative and qualitative evaluation which demonstrates the robustness of our method to track people in environments where object features are not clear and have similar colour to the background. We show that our approach achieves excellent performance in terms of the multiple object tracking accuracy (MOTA) metrics, and that it is particularly robust to initialisation differences when compared with baseline and state of the art trackers. Using the Online Tracking Benchmark (OTB) videos we also demonstrate that our tracker is very strong in the presence of background clutter, deformation and illumination variation.

## 1. Introduction

### 1.1. The Need for Meeting Support Technologies

Meetings are important events in any organisation. They are essential for knowledge sharing, knowledge creation, information exchange and informed decision making. For a variety of reasons, people may not be able to attend a meeting or may miss important information even if they do attend. Note taking is one of the possible solutions; however, this can be subjective and inaccurate, as well as laborious. To address this problem, a meeting recorder is needed to enable future reviewing.

Meeting recording and understanding has attracted much attention via research on a diverse range of technologies. These technologies range from physical capturing to meeting analysis and semantic processing. Meeting capture records different types of data needed for meeting analysis such as video, audio and textual data. Meeting analysis is a low-level processing layer used to analyse the captured data from the meeting capture module, while semantic processing is a high-level layer which is responsible for handling semantic manipulation such as browsing [1].

The analysis of human activities inside meeting rooms has received significant interest in the literature, and has been the focus of a number of international projects attempting to develop prototypes for intelligent meeting rooms [2]. Projects have included the Multi Modal Meeting Manager (M4) project [3,4], the International Computer Science Institute (ICSI) project [5], the Augmented Multi-party Interaction (AMI) project [6], the Computers in the Human Interaction Loop (CHIL) project [7], and the Cognitive Assistant that Learns and Organizes (CALO) project [8].

The analysis of human activities inside meeting rooms is one of important cues and can be used to automatically annotated video/meeting browser. For example, the user will be presented with key events such as when a specific attendee went to the white-board, or when all participants are sitting and one at white board, the presentation event was detected, or when attendees were in animated discussion-thereby allowing the user to jump to these key events when viewing the video. Since the end of the 1990s, much research has been carried out on the automatic recording, recognition and interpretation of meetings and many smart meeting systems have been published in the literature. An early smart meeting system was introduced in 1998 [9,10,11]. The meeting room system consists of many components, which include a camera and microphone to record the meeting, software for speaker identification, speech recognition, and dialogue summarisation for the recognition functions and a meeting browser to allow the user to search and browse a meeting. The Distributed Meetings (DM) system was developed by Microsoft. It consists of an omnidirectional camera and microphone array designed for capturing meetings. Its recognition module consists of sound source localisation and person detection and tracking. A meeting browser enables users to choose a preferred view of the meeting room (e.g., all meeting participants). Furthermore, this system supports meeting broadcast and future reviewing [12]. A portable meeting recorder was proposed in Reference [13]. It is a portable device consisting of an omnidirectional camera, with four microphones at the corners. Firstly, the audio is processed to detect the location of the speaker. Recognition modules such as face extraction, motion analysis and audio activity are used to generate meta data, which is then presented in the meeting browser. The meeting browser allows users to browse meetings by listening to chosen speakers, searching meeting transcripts and viewing presentation slides. An intelligent meeting room called the AVIARY system was developed by the University of California, San Diego. Two PC computers are used—one is used to perform 3D tracking from four static cameras. The tracking results are then sent to another PC which handles all other functionalities, such as person identification, event recognition and best view camera selection, which can be browsed through a graphical user interface [14,15]. CAMEO, the Camera Assisted Meeting Event Observer, is an omnidirectional system consisting of several cameras to generate a panoramic view. CAMEO is a physical system to recognise people’s actions in a meeting and then generate a summary of events occurring in the meeting [16]. A meeting application was proposed in Reference [17]. It consists of four personal cameras and one camera to capture a room overview. Audio analysis and face direction are used to recognise important parts in a meeting. A meeting browser also enables users to browse the current status of a meeting and select meeting contents according to user preferences. A new smart meeting system was recently presented in Reference [18]. It records the meeting using two stationary cameras, two PTZ cameras, one ceiling panoramic camera, two separate microphones and three microphone arrays. Sound source localisation and people tracking are applied to monitor and automatically register meeting participants. A meeting report of participant faces, along with recordings of their presentation recording, is then generated.

An essential building block for scene analysis is the detection and tracking of objects in the scene. The biggest challenge facing visual tracking systems in indoor environments (e.g., meeting scenarios) is still the reliable detection of persons when their appearance changes from different camera views, with partial occlusions occurring in natural uncontrolled environments [19,20,21].

### 1.2. Meetings

Object detection and tracking is an important problem with a wide range of applications including robotics, video surveillance, autonomous driving vehicles and intelligent systems (i.e., smart meeting rooms). We focus on meeting analysis for several reasons.

Meetings are the milestones for many people to schedule and do their work. In collaborative decision-making, the meeting is a place where group goals and work are shaped. Understanding what happens in meetings is very useful whether the aim is to expose the meeting’s content or simply to identify where the process of a group can be improved. Thirdly, most organizations work globally, in the face of business fragmentations, and this presents difficulties for an organization to maintain and access the institutional memory which they need to make good decisions. It is difficult or even impossible to document everything. Therefore it is economically important to control important information in meetings ranging from recording, analysis to access the content. Finally, many organizations record and archive their important meetings, even though they lack decent tools to review these records later. Improving this technology effectively bring benefits to an organization. People cannot always attend all meetings that affect them, therefore accessing their content efficiently is likely to help [22] (popescu-Belis and Carletta, 2012).

In this paper, we describe a novel people tracking-by-detection algorithm to track meeting participants from an overhead camera. The rationale of using an overhead camera is that all objects can typically be seen clearly, with much less occlusion than would be seen in normal views. However, using an overhead view for people detection also poses challenges such as radial distortion of the image, cluttered backgrounds, and a lack of distinctive colour or consistent features [23].

Due to a lack of clear image features available when tracking people from overhead, we focus our approach on movement rather than feature tracking. The standard techniques for movement based tracking (e.g., background subtraction and frame differencing) however require background models, and they struggle when objects exhibit varying movement speeds (e.g., with periods of no movement and periods of fast movement). Often slow moving objects cannot be successfully omitted from the background model, nor be detected by traditional consecutive frame differencing methods. To address this problem, we propose a coarse-to-fine algorithm using a combination of a novel adaptive accumulated frame differencing (AAFD) technique and Shi-Tomasi corner detection. This coarse-to-fine approach allows robust blunder recovery, since each video frame is considered separately by the AAFD step.

Firstly, the region of movement is extracted using AAFD, and then corner detection is applied on this region. These corners are further processed and only corners which coincide with movement are considered, thereby rejecting the myriad of corners that are due to static objects in the environment. Our goal is to combine techniques that use totally different evidence (i.e., one is temporal while the other is spatial), while working in an environment with no prior knowledge and in which meeting participants may be moving very little (e.g., while seated) or relatively fast (while walking).

## 2. Related Work

We consider related work on people detection and tracking methods in two ways—firstly, application based approaches (i.e., smart meeting systems), and secondly, object detection and tracking methods (i.e., image processing algorithms).

### 2.1. People Detection and Tracking in Meeting Applications

Numerous researchers have worked on vision-based participant tracking in meeting environments using single or multiple cameras, standard video cameras or omnidirectional cameras.

#### 2.1.1. Tracking Approaches Using Single Video Cameras

A number of distinct approaches to tracking people from a single video sequence have been proposed. Hradis and Jurnek [24] describe a multiple head tracker, where the tracking task is divided into two parts. Firstly, the head is detected based on a skin colour model, background subtraction and component analysis. Secondly, a Kanade-Lucas-Tomasi (KLT) feature tracker combined with a colour model is used to track detected objects.

Nait-Charif and McKenna [25] propose a reliable head tracking algorithm, evaluated on the PETS-ICVS 2003 video data sets. Firstly, the head is modelled using a fixed ellipse that represents the head boundary and the ellipse’s interior region is represented using a colour histogram. This head likelihood model is used by a particle filter to track participants in the meeting room. The tracker is initialised based on manually defined room layout constraints.

Wu and Nevatia [26] developed a head-shoulder detection algorithm which was applied to track multiple people in a meeting room. The object is tracked by head-shoulder detection in every frame; when the detection is not found mean shift tracking is applied.

Li and Nevatia [27] addressed the problem of recognising, localising and tracking multiple objects in meeting room videos. Object level spatio-temporal relationships were incorporated into their framework. The contextual relationships are modelled using dynamic Markov random fields. The human is defined as a key object which is detected first, and is used to provide contextual guidance for detecting other objects (e.g., tables, paper, computers). The cascade human upper body classifier is used to detect key objects. The other object categories are detected based on feature point classifiers (Corner points, SIFT features). This approach relies on an appearance model, and a stronger model is needed for a good performance.

Each of the techniques considered in this section were applied to a variety of frontal camera configurations, and therefore cannot be deployed on the overhead view, on which the current research focuses, due to different nature of evidence in these images.

#### 2.1.2. 3D Vision-Based Tracking Algorithms

As part of the Computers in the Human Interaction Loop (CHIL) project, a set of 3D tracking methods was developed by a consortium involving the Interactive Systems Labs of the University at Karlsruhe (UKA), the Research and Education Society in Information Technologies at Athens Information Technology (AIT) and the Universitat Politecnica de Catalunya (UPC).

The contribution from UKA involves a multi-view tracker, which uses several fixed cameras installed at the corners of the meeting room. The tracking is performed on each camera image, then the information is combined to produce a 3D hypothesis of a person’s position. Detection is performed firstly based on background subtraction to find the region of movement, followed by a cascades classifier to detect a person. After that, the tracker is initialized based on a colour histogram for the detected person. The tracking is performed based on colour features using the mean shift tracking algorithm [20].

The contribution from UPC involves adaptive background subtraction applied on each camera view, followed by 3D blob reconstruction based on a combination of foreground segmentations from each separate view. Following this, each blob is modelled based on three features—the velocity, the volume and a histogram of colour values. Kalman filtering is used to update the object’s velocity and its size. Finally, assignments are achieved using weighted Euclidean distance to match a blob with the identified template features [28].

The contribution from the AIT site involves a 3D video tracker combining the evidence from multiple 2D video trackers. The 2D video trackers consist of three modules—adaptive background, measurement and Kalman filtering. The measurement module performs the association between the foreground pixels and targets. It also initializes a new target for non-associated foreground segments. Additionally, it manipulates the merging or splitting of targets based on an analysis of foreground objects. Finally, the object information is sent to the Kalman filtering module to update the object’s position, velocity and size. This information is also sent to an adaptive background module to assist the adaptive background subtraction process [29].

#### 2.1.3. Tracking Approaches Using Omnidirectional Cameras

As omnidirectional images produce a wide view with low cost, a number of tracking algorithms have been developed using omnidirectional views.

A fast detection and tracking algorithm from panoramic images, referred to as CAMEO (the Camera Assisted Meeting Event Observer) is proposed in Reference [16], and involves a system to recognise people’s actions, followed by the generation of a summary of events occurring in the meeting. CAMEO’s tracking system consists of a number of components—a region of interest (ROI) extractor, a face detector, a shape detector, a mean shift colour tracker, and a Bayesian network-based action recogniser. The location of the face is tracked, with colour information providing robustness to rotation and partial occlusion. A colour histogram is calculated in the frame where the face has been detected and a data histogram is computed from a candidate location in a successive frame. Similarity is measured using the Bhattacharya coefficient.

A tracking algorithm for meeting rooms using omnidirectional views presented by Wallhoff et al. [30] also relied primarily on colour, in this case to detect both head and hands. This work used a neural network to detect the face, and a particle filter to track the face in image sequences.

The UKA site’s “top view” person tracking approach was implemented in Reference [20] as part of the CHIL project. This approach operates on images captured from a fisheye camera installed in the ceiling of the meeting room. The tracking algorithm consists of two stages—foreground extraction based on an adaptive background model, followed by a blob assignment algorithm. The latter algorithm performs an optimal assignment between detected blobs and a set of person models incorporating position, velocity and radius.

This section explored the state-of-the-art people tracking methods used in smart meeting rooms. Most of the existing tracking methods use a set of multiple cameras to track meeting participants. Recognition rates for tracking methods are sometimes not robust in real-world settings; this a reason underlying why smart meeting systems are not widely used. Therefore, the current technologies must be improved to a level that is robust and usable. The most significant challenge facing visual tracking systems in an indoor environment (e.g., meeting scenarios) is still the reliable detection of persons when their appearance changes from different camera views, with partial occlusions occurring in natural, uncontrolled environments [19,21]. According to the evaluation results presented by the CLEAR 2006 workshop for visual tracking tasks, a tracker which used a single input video stream from a top-view camera was the best-performing tracker compared with other approaches based on the fusion of multiple camera streams [31]. The rationale of using an overhead camera is that all objects can be typically seen clearly. However, using an overhead view also poses many challenges, such as a lack of distinctive features.

The next section will present a literature review of motion-based detection methods which provide a robust approach to detect and track people in this type of an environment.

### 2.2. Object Detection and Tracking

Object tracking is the task of estimating object motion in video frames. Any tracking algorithm needs an object detection method applied in every frame or when an object first appears in the video. Various object representation methods are proposed. We focus on motion-based detection methods, as motion features are one of the most robust features in environments with major changes in object appearance. The first Section (Section 2.2.1) reviews various motion detection approaches proposed in the literature. The second Section (Section 2.2.2) reviews visual object tracking with a focus on online object tracking.

#### 2.2.1. Object Detection Based on Motion-Based Methods

Moving object detection is the first and most important step in video analysis. This is the process of separating foreground objects from the background [32]. Assuming a stationary camera is used, the object motion plays a key role in video-based foreground/background separation [33]. In recent years, various approaches for moving object detection have been proposed, within various application domains. Firstly, we give an overview of the several motion detection methods based on background modelling. Then, the other motion detection methods developed in the literature, such as optical flow and frame differencing are reviewed.

##### Motion Detection Methods Based on Background Modelling

Background subtraction is a technique commonly used to detect moving objects [34,35,36,37]. This involves firstly building a background model using a set of images, and then calculating the difference between the current frame and the background model, in order to detect the objects of interest. The background must be modelled accurately, with frequent updates to consider changes in the background, such as changes in lighting conditions, scene geometry or moving objects (e.g., trees shaken by the wind) [38]. Background subtraction methods can be categorised into parametric, nonparametric or predictive techniques [39].

Parametric models use a single probability density function to model a background at each pixel location. One of the most famous parametric background methods is the Gaussian model. For example, Wren et al. [40] proposed a Running Gaussian average, where the background is modelled at each pixel location with a single Gaussian distribution. However, a single Gaussian model cannot deal with dynamic background changes (e.g., tree leaves animated by the wind) due to its low updating rate of the background model [41]. In order to cope with these dynamic background changes, Stauffer and Grimson [42] proposed Gaussian mixture modelling (GMM), which is one of the most common adaptive background modelling techniques, whereby each pixel is modelled as a mixture of Gaussians and the background model is updated to cope with scene changes. However, this technique remains challenging for slow-moving objects, and it is particularly sensitive to speed changes [43]. Furthermore, the adaptive Gaussian mixture background model assumes that the background model can be initialised using a set of training frames in the absence of foreground objects, which is frequently difficult to achieve [44].

Parametric models cannot handle a complex scene when a background has rapid variations, as it is difficult to accurately model the background with only a few Gaussian models. Kernel density estimation (KDE) is an example of a nonparametric background model which estimates the background probabilities at each pixel intensity from many recent samples over time using KDE [45,46]. Barnich and Droogenbroeck [47] proposed a pixel-based nonparametric background model, namely the Visual Background Extractor (VIBE), which models the background via a set of sample values as a pixel model using a novel random selection strategy. VIBE achieved a good level of performance; however, the pixel model is initialised based on values found in the spatial neighbourhood of each pixel; therefore, the presence of foreground objects at the first frame will cause ghosting in foreground detection.

Predictive techniques (estimation filters) make predictions about background pixels in the next image. The Wallflower system developed by Toyama et al. [48] estimates the background model using the Wiener filter at the pixel level. The Wiener filter is a linear predictor based on the recent history of values. A pixel is considered as foreground when it deviates significantly from its predicted value. Then, homogenous regions of foreground objects are filled at the object level, and a frame-level component is added to detect global illumination changes. The Running average filter is another example of an estimation filter which estimates the background recursively using an infinite impulse response (IIR) filter [49].

##### Other Motion Detection Methods

The background subtraction methods described in the previous section have been used widely. However, other methods which use other types of modelling have been developed to detect moving objects such as optical flow and frame differencing. Optical flow refers to the flow vectors of moving objects, and it indicates the speed of movement of pixels in subsequent frames. It indicates the velocity and the direction of pixel movements [39,50]. Han et al. [51] combined optical flow with three-frame differencing to detect moving objects. Optical flow can detect objects with no prior knowledge; however, it does not perform adequately when objects are moving slowly.

Traditional frame differencing captures the change between two consecutive frames by calculating the absolute difference between the two frames [52]. Most of the existing frame differencing methods [53] extract moving objects using a single temporal scale [54]. Therefore, frame differencing can only detect fast-moving objects, while slow moving objects will not be detected.

Little work has been done to detect objects which exhibit both slow and fast movements. Chinchkhede and Uke [55] proposed an image segmentation algorithm using background subtraction and expectation maximisation (EM), where each pixel is classified, then the slow-moving object is located, and its “shadow” is distinguished from the moving object using modified background subtraction. A combination of background subtraction and frame difference is used to address the problem of incomplete detection for the moving object [56].

Cumulative frame differencing (CFD), which involves the summation of successive frame differencing, of presented by Alsmadi et al. [39] to improve the detection of slow-moving vehicles. Firstly, the successive frame differences are accumulated to generate cumulative frame difference (CFD). Then, to determine a foreground pixel, a dynamic threshold value which, is based on the standard deviation of the absolute cumulative frame differencing (CFD), is calculated. Finally, when CFD for a pixel greater than or equal the estimated threshold, the pixel is considered foreground.

A motion history image (MHI) keeps a history of temporal changes at each pixel location, which then decays over time, whereas more recent moving pixels are brighter. This approach has been used to build a motion template for human action recognition [57] and was used by Morde et al. [58] for stationary object detection. The robustness of the basic MHI method depends on the motion detection algorithm (e.g., background subtraction, frame differencing or optical flow) used to identify motion pixels, which is therefore used to update MHI [59].

Most of the existing methods do not have the required adaptability to detect and analyse moving objects using different temporal scales. Leng and Dai [52] presented an adaptive accumulated frame differencing approach to extract objects from head and shoulder video sequences. Firstly, the frame difference (FD) is divided into blocks (8 × 8 pixels). Then, the sum of FD within a block is calculated and used as a criterion for motion analysis. For each block, FD is accumulated using a different number of frames, based on its motion attributes. Finally, thresholding and post-processing are applied to segment the object. Zhang et al. [54] used spatial and temporal domains to detect and track multiple objects. They proposed a spatial temporal detection and tracking algorithm which calculates temporal-spatial windows for each object using octree decomposition of the temporal-spatial domain. This works well on moving platforms with uniform linear motion. However, cases where the object is moved in arbitrary directions, as well as cases where the object is hidden in the scene, are not considered.

There is another set of literature focusing on the detection of stationary foreground objects (i.e., foreground objects which remain static for several frames). According to Cuevas et al. [60], stationary foreground objects can be classified into three types:

- Type A: insertion of the stationary moving object into the scene by a human, such as a backpack or suitcase.
- Type B: insertion of a moving object that has become static without any interaction with a human (e.g., a vehicle that has been parked).
- Type C: a moving person that becomes totally or partially static.

Most of the published strategies for stationary object detection focus on the detection of abandoned objects (i.e., type A), and there are no strategies that focus only on the detection of partially static objects (i.e., type C). Most of these methods use a background subtraction algorithm as the first step to obtain the foreground mask, followed by other algorithms to detect stationary objects. For example, an analysis of the persistence of the pixels that is classified previously as foreground is used to detect stationary objects. That is when a pixel is classified as foreground across several frames; this pixel is considered part of a stationary foreground object [61]. In Martínez et al. [62], static moving objects (e.g., people in offices or vehicles on urban roads) are detected by building three background models with three different buffers. Then, the detection results are used by a finite state machine to detect when a moving object becomes static and when a static object moves again.

#### 2.2.2. Object Tracking

Object tracking, which aims to estimate the position of a target in every frame of a video sequence, is a fundamental problem in computer vision. Many factors, such as illumination variation, scale variation, occlusion, deformation, background clutter and low resolution, add numerous difficulties to the tracking problem [63].

Generally, object tracking algorithms consist of a motion model and appearance model. The motion model predicts the approximate location of an object. The appearance model can then evaluate the presence of the object of interest at a particular location [64]. We focus on the tracking methods that build appearance models to discriminate an object from the background. The current appearance tracking algorithms can be categorised into either generative or discriminative approaches. In generative trackers, the tracking task is carried out via searching for patches which are most similar to the target. The discriminative tracker performs tracking by separating the object from the background [65].

##### Generative Tracking Methods

Generative tracking methods model the appearance of the object. Template tracking is a typical approach. A target template (an image patch or colour histogram) is used to describe the object, then the position that minimises the mismatch error between the target template and candidate template is considered to be the object’s new position [66]. Template matching can be static (e.g., Mean shift tracking) [67] or adaptive (e.g., Lucas-Kanade optical flow) [68].

Mean shift tracking uses the target colour histogram formed in the first frame as a target template without any update during the tracking. For each new frame, the Bhattacharyya metric is used to compare two histograms of candidate windows and the target template. Then, to find the best new object location, a mean shift is applied to find the mode of a function that maximises the Bhattacharyya metric [67,69].

Optical flow initially requires the detection of corner points. A corner in an image is defined as a point at which there is a large variation in intensity in the neighbourhood of this point in all directions. Harris Corner Detection [70] and Shi-Tomasi Detection [71] are the most common corner detection methods. These detected corners are then tracked using optical flow techniques such as Lucas-Kanade [68]. The optical flow attempts to match corners in the previous frame to the new frame in order to track an object due to the motion of that object between two frames. Optical flow works based on the assumptions that, while the object changes its position from frame 1 to frame 2, pixel intensities do not change substantially between consecutive frames. The Lucas-Kanade method estimates a movement vector by comparing two consecutive frames and assigning the movement vector for every pixel of interest in the scene [72].

Generative tracking methods need an effective appearance model. The generative trackers model only the appearance of the object, and they often fail in an environment when an object’s features are similar to the background (e.g., in a cluttered background) [66]. In order to address this problem, recent trackers employ the information from the object and background- these are discriminative methods.

##### Discriminative Tracking Methods

In recent years, it has been found that background information is very important for effective tracking [73,74], which suggests that discriminative tracking methods are more competitive, and often achieve superior results [64]. A common approach adopted for discriminative trackers is to build a classifier which is trained online and which aims to distinguish between an object and its background. The online updating strategy alleviates significant appearance changes, cluttered backgrounds and tracking drift. Numerous classifiers have been adapted for object tracking, such as Multiple Instance learning boosting trackers (MILs) [64] and tracking-learning-detection (TLD) [66].

The MIL tracker uses a track-by-detection approach. It is based on multiple-instance learning instead of traditional supervised learning methods. The basic design of the MIL tracking algorithm involves the following:

- Firstly, at every time step, t, the object location is known by the tracker. Furthermore, the tracker crops out a set of image patches inside the region of interest (within a radius, s, of the current tracker location), and feature vectors are calculated.
- Secondly, the MIL classifier is used to calculate the probability of each image patch being foreground.
- The tracker location is updated based on the image patch with the highest probability.
- A set of positive and negative versions of image patches is cropped, and the MIL appearance model is updated with one positive bag and a number of negative bags (each bag containing a single negative image patch).

Recently, a comprehensive evaluation of online tracking algorithms was performed by Wu et al. [73,74]. In this evaluation, the TLD tracker was one of the top 10-performing trackers. The TLD performs long-term tracking in a video stream. It consists of three components—Tracker, Detector and Learning. The tracking component predicts an object’s location based on its motion estimation between successive frames. The detector performs the scanning of the image and classifies each patch to be accepted or rejected based on object presence. The learning module initialises the detector at the first frame, estimates the detector error and generates positive and negative examples to update the object detector. Finally, the integrator combines tracker and detector bounding boxes into one bounding rectangle as output for the TLD tracker [66,75].

Another common approach of discriminative trackers is based on correlation filters. The basic idea of correlation filter tracking is firstly to estimate the optimal image filter by training the filter using multiple shifted instances of the object at the first frame. Then, at each new frame, the filtering image is tested with an input image to find the object location. The output is a Gaussian shape centred at the target location, where the output will be 1 near the object location, decreasing with distance [76].

The Kernelized Correlation Filter (KCF) [77] tracker was one of the top 10- performing trackers in online tracking algorithm evaluation [73,74]. The tracking pipeline of the KCF tracker is as follows:

- In the first frame, the model is trained with an image patch obtained based on the initial position of the object.
- For a new frame, a test image is extracted based on the current location of the bounding box. After that, the target is detected by finding the maximum score location and updating the target position (bounding box location).
- Finally, a new model is trained based on the new location [77].

Henriques et al. [78] replaced the grey scale template with a histogram of oriented gradient (HOG). The KCF is extended to extract colour features from an image patch instead of a grey scale image patch for filter learning. It achieves the best-performing tracker on background clutter (BC), deformation (DEF), illumination variation (IV) and occlusion (OCC) tracking challenges in the online tracking benchmark (OTB) [63].

The discriminative correlation filter with channel and spatial reliability CSR-DCF proposed by Lukežič et al. [79] to address issues with discriminative correlation filters (e.g., using a rectangular bounding box allows the background to be included as part of the filter’s learning). The CSR-DCF tracker improves discriminative correlation filter trackers by adding two features—spatial reliability and channel reliability. A spatial reliability map is estimated using colour segmentation to find pixels belonging to the target in the training region. This improves the filter learning step (update) to only consider foreground pixels. The channel reliability score reflects the quality of each filter channel in object localisation. The CSR-DCF tracker uses histogram of gradients (HOG) and colour-names features. The CSR-DCF tracker achieves state-of-the art results with regard to the visual object challenge (VOT) (http://www.votchallenge.net/), specifically VOT 2016, VOT 2015 and OTB100 [74], and it is one of the top-performing trackers in VOT 2017.

## 3. Combining Accumulated Frame Differencing and Corner Detection for Motion Detection

We propose a novel motion detection algorithm which combines temporally-adaptive accumulated frame differencing with shape features using Shi-Tomasi corner detection [70,71]. Frame differencing data is a certain type of evidence describing how much pixels are changing over time, whereas corner detection is completely different evidence which searches for discontinuity in the pixel value or the edges, considered in a single frame. These are totally different forms of evidence and therefore are appropriate to use in combination.

Our approach differs from previous work in two major ways—firstly, in its use of accumulated frame differencing using different temporal window sizes based on analysis of movement shape features. For example, a large temporal window size is used to detect slow moving objects (characterised by small, compact movement shapes) and a small window size is used to detect fast moving objects (characterised by large, elongated movement shapes).In many real-life scenarios, moving objects vary in their speed and perceived size, therefore a motion detection method is needed which can detect objects regardless of whether their motion is significant or small. Existing methods do not have the adaptability to detect and analyze moving objects using different temporal scales.

The second novel contribution in our approach is that we propose coarse-to-fine track-by-detection algorithm which does not rely on prior knowledge; therefore, the overall system can recover from blunders. Recently, discriminative object tracking achieved superior results to track objects of interest. However, a discriminative tracker is prone to drift in the case of severe interferences, such as deformation, background clutter, scale variation and occlusion. The online updating step updates the tracker based on the predicted object location. If the predicted location is not precise, the performance of the tracker may degrade over time and cause drift problems. Our coarse-to-fine approach is proposed in order to address this drifting problem.

### 3.1. Outline of Combination of AAFD and Corner Detection Technique

**Figure 1:**
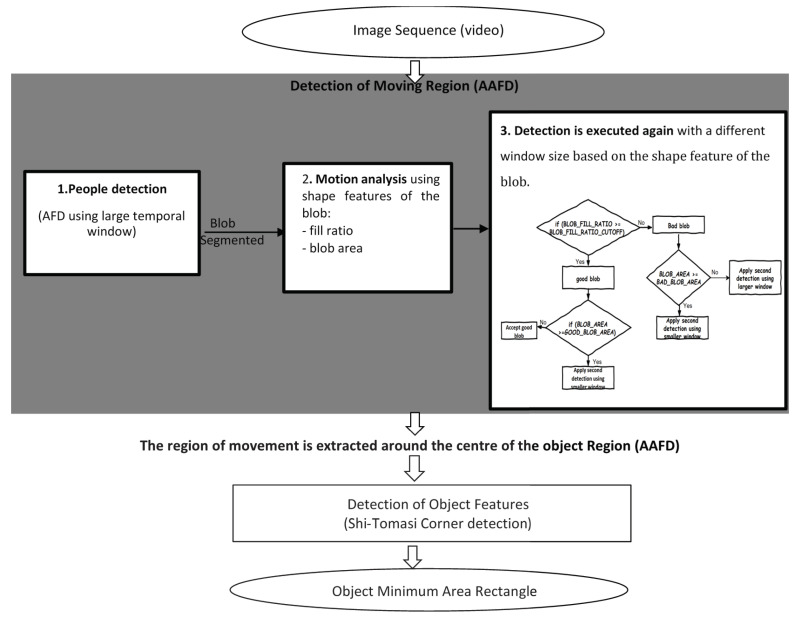
Block diagram of combination of Adaptive Accumulated Frame differencing (AAFD) and corner detector for people detection.

Figure 1 provides an outline of our overall algorithm. We use standard morphological techniques to extract blobs (regions of connected pixels) from accumulated difference images; these are assumed to indicate coherent moving objects. Various phenomena in meeting videos may cause objects to appear as split or merged blobs, and therefore additional processing is required. Corner features are detected and further processed to yield moving corner points. Finally, the minimum area rectangle fitting these corner points is found. Our approach is further described below.

#### 3.1.1. Detection of Moving Region

The region of movement is extracted using AAFD, where object motion is segmented adaptively based on an analysis of motion shape features (i.e., object shapes in the accumulated difference images). A large temporal window size is used to accumulate frame differencing data for slow moving objects, while fast moving objects are segmented using a small temporal window. Once the object is detected, the region of movement is extracted around the centre of the object.

Our algorithm performs detection of people based on three stages:

1. People detection is applied based on an accumulated frame differencing image using a large temporal window size(e.g., Temporal window size = 100). Starting with a large window size allows the robust segmentation of all foreground pixels, even if they belong to objects moving very little.
2. For each detected blob, motion analysis using the shape features of the blob is applied. Two shape features, namely “fill ratio” and “blob area”, are used to accept or reject blobs. Fill ratio refers to the area of the blob divided by the area of its bounding box. Acceptable blobs are assumed to be quite square and their fill ratio will be closer to 1 than 0. Fast moving objects lead to elongated blobs with a low fill ratio. If the fill ratio is smaller than a defined threshold, we conclude that the blob relates to the either the merging of multiple nearby objects, or to a fast-moving single object. With a large temporal window size, blob area is therefore a suitable feature for rejecting small blobs (due to noise) and large blobs (due to merged or fast moving objects).
3. Finally, the detection is executed again with a different temporal window size based on the shape feature of the blob,(e.g, larger temporal window size = 150, small temporal window size = 25).

##### Lossy Compression Issues

Unfortunately, lossy compression artefacts are amplified during AFD image calculation; this noise appears as white blocks in the difference image. We use a coefficient of motion calculated for the region of interest (ROI) to reduce these compression artefacts, as follows:

1. Firstly, an ROI(x,y) is converted to a binary image:
  (1) $$ROI_{b}\left(x,y\right)=\left\{\begin{array}{cc} 255, & ifROI(x,y)>thresh\\ 0, & otherwise \end{array}\right.$$
2. Secondly, the coefficient of motion is calculated =
  (2) $$\frac{\mathrm{Number}\;\mathrm{of}\;\mathrm{non}\;\mathrm{zero}\;\mathrm{pixels}}{\mathrm{total}\;\mathrm{number}\;\mathrm{of}\;\mathrm{pixels}}$$

**Figure 2:**
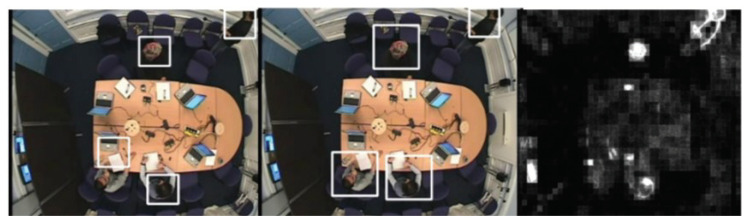
Examples of region of movement extraction with/without application of our motion coefficient (Frame 4921 of the ES2002a video from Augmented Multiparty Interaction (AMI) video data sets), where the first image is region of movement extraction without using motion, the second image is region of movement extraction based on coefficient of motion and the third image is accumulated frame differencing with window size = 100. Using this coefficient of motion reduces errors resulting from compression artefacts.

When the coefficient of motion is greater than a threshold value, the region of movement is extracted based on AAFD. Otherwise, the region of movement is extracted based on data from the previous image frame. Figure 2 shows the effect of this approach.

#### 3.1.2. Detection of Object Features (Shi-Tomasi Corner Detection)

The N strongest corners are found by applying the Shi-Tomasi corner detection method in the region of movement extracted based on AAFD. Firstly, all corners below the threshold value are rejected (The Shi-Tomasi Corner Detector requires that the minimum eigenvalue should be above a threshold value. A parameter called quality level is used to calculate this value. The threshold value is calculated as—quality level × maximum eigenvalue through the image. Quality level is set to 0.01). Then, the remaining corners are sorted in descending order based on their minimum eigenvalue. The strongest corners are returned, then, based on the specified minimum distance between corners, nearby corners are rejected, and the N (specified maximum number of corners) strongest corners are returned [71,80]. 

##### Combining Corners with Motion

**Figure 3:**
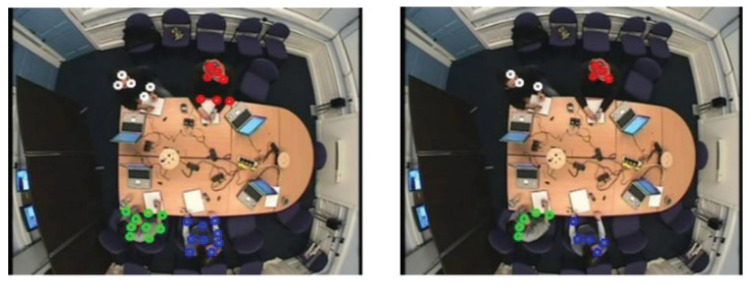
Sample images to show corner detection; **left**: without a corner scoring function; **right**: with a corner scoring function.

As our goal is to track meeting participants, we are interested in the detection of motion corners (i.e., corners on foreground objects only). In order to identify motion corners, we use a scoring function to assign a score value for each corner point based on its corner strength combined with AFD evidence at that point. For all N corner points $C_{N}\left(x,y\right)$, the score S(N) is defined as:

(3) $$S\left(N\right)=C_{N}\left(x,y\right)*FD_{N}{\left(x,y\right)}^{x},$$

where $C_{N}\left(x,y\right)$ is the minimum eigenvalue and $FD_{N}\left(x,y\right)$ is AFD data which ranges from 0 (black) to 255 (white), and *x* is an empirically determined constant and in our experiment *x* = 0.2. Figure 3 shows an example of using this score function to reduce background corner points.

To consider only the motion corners, all corners are sorted in descending order based on their score value. Then, the highest N corners are returned and the minimum area rectangle fitting them is calculated to update the centre of the region of interest. This is further detailed in Reference [81]-note that this earlier report reflects our incomplete work-in-progress and lacks the detailed analysis and testing of the current paper.

## 4. Results and Discussion

We experimentally evaluate our method in two contexts—firstly, using a public dataset of meeting videos and the multiple object tracking (MOT) metrics, and secondly using per-attribute analysis with a general (non-meeting) data set and the online tracking benchmark (OTB).

Section 4.1 presents experimental results and analysis of our method using the Clear-MOT metrics which were used to evaluate the people tracking methods developed during the CHIL project. We also discuss evaluation results based on widely used general-purpose evaluation metrics for multiple object tracking. We compare our results to those published during the CHIL project as well as to modern state of the art trackers. Section 4.2 reports on the test analysis of our method using a recent visual object tracking dataset based on specific challenging attributes (i.e., background clutter, illumination variation, deformation, scale variation and occlusion).

### 4.1. Performance Evaluation in a Meeting Context

#### 4.1.1. Data Set

**Figure 4:**
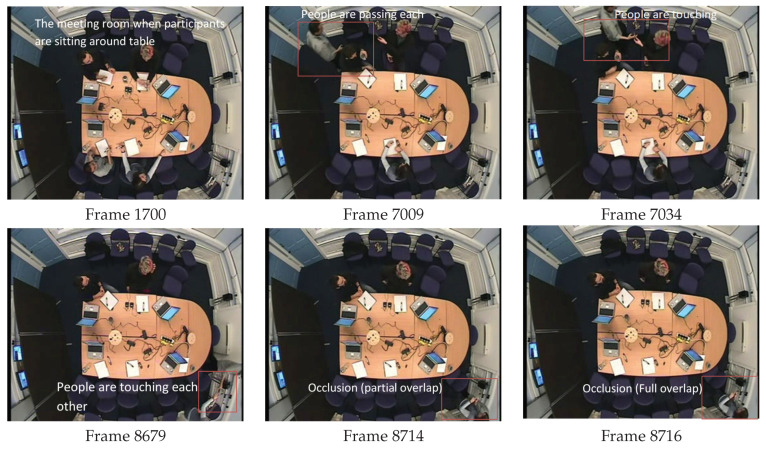
Example from ES2002a video. Frame 1700 shows the meeting room when participants are sitting around the table. Frame 7009 is an example of the challenge when people are passing each other. Frames 7034 and 8679 show touching cases. Examples of occlusion (overlapping) are shown in frames 8714 and 8716.

The Augmented Multiparty Interaction (AMI) public meeting corpus is used to evaluate the performance of our proposed approach. Testing was performed using ES2002a video [6]. This video was recorded from the overhead camera in a meeting room with four meeting participants. Meeting participants are sitting around the table and perform general meeting actions such as sitting down, standing up, moving to the whiteboard and discussion. The video contains many detection and tracking challenges—examples include people sitting near each other, touching, people walking past each other, as well as variations in size and occlusion—see Figure 4. Testing was done by dividing the video into sequences which exhibited specific tracking challenges—see Table 1.

#### 4.1.2. Evaluation Methodology

The performance of our detection and tracking algorithm can be evaluated visually (qualitatively) and quantitatively.

##### Qualitative Evaluation

**Figure 5:**
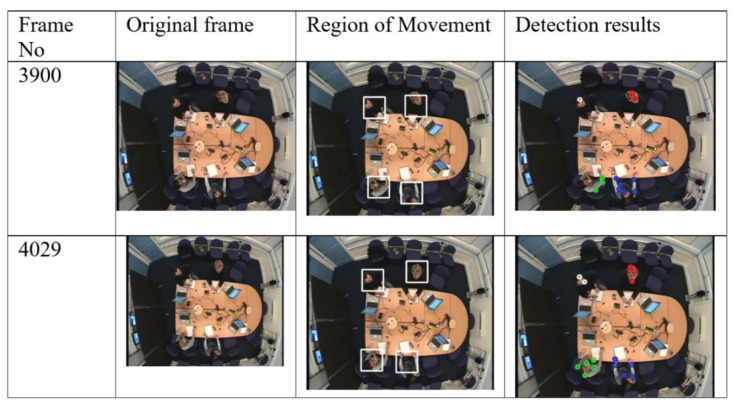
Detection results for slow movement (i.e., people are sitting). Left to right, frame number, original frame, region of movement, detection results.

**Figure 6:**
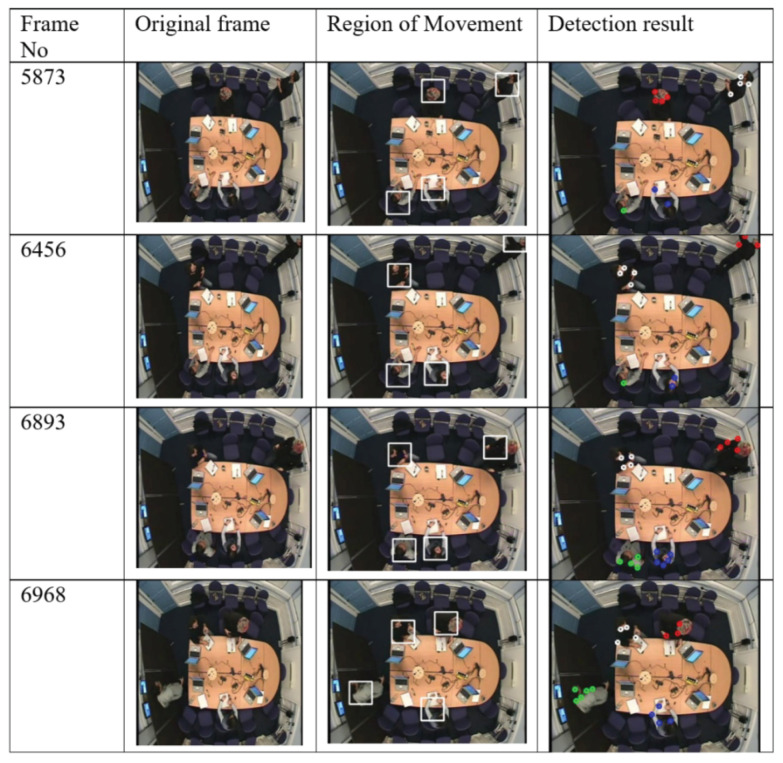
Detection results for different motions of people (i.e., low motion when people are sitting and significant motion when they are moving in a meeting room).

**Figure 7:**
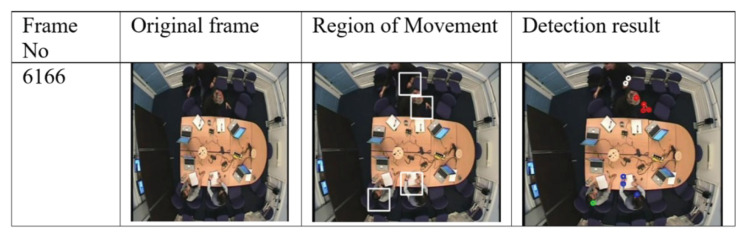
Sample image to show detection result when meeting participants are near each other.

Visual evaluation can be done by displaying a video of images where a rectangle is drawn around each person [38]. The performance of our detection and tracking algorithm has been evaluated visually (qualitatively). As depicted in Figure 5, Figure 6 and Figure 7, the qualitative result highlights a good performance of the proposed algorithm in different scenarios.

Figure 5 illustrates the detection result using our algorithm which shows a slow movement scenario as all people are sitting around the table making very small motions. The first column provides the original frame, the second column shows the result of region of movement extraction using AAFD and the last column presents the final detected corner points.

Figure 6 demonstrates the case where there is varying amounts of motion. For example, in Frame 5873 and Frame 6456 one person is standing at the white board while others are sitting. The person starts moving to the whiteboard in Frame 6968. Frame 6893 shows a person returning to his seat and another one starting to move to the white board.

Figure 7 shows an example of detection when people are near to each other.

##### Quantitative Evaluation

Although multiple object tracking is an active research area, there is a lack of common metrics for measuring the performance, which makes the quantitative comparison of different tracking approaches difficult [82]. To remedy this, several common (standard) quantitative measurements for multiple object tracking have been proposed in the literature [83,84].

Clear-MOT evaluation is a common standard evaluation tool widely used for multiple object tracking (MOT) [83]. It was used to evaluate the performance of people tracking in the CHIL meeting project; see Section 4.1.3.

Track quality measures are common measures used to evaluate the performance of entire trajectories for multiple object tracking [84].

#### 4.1.3. Tracking Evaluation Results Using Clear-Mot Metrics

##### Test Objective and Parameters

The objective of our proposed coarse-to-fine people detection and tracking algorithm was to find people’s movements in the AMI meeting videos robustly. The scenario was that of meetings using the overhead camera. The method was evaluated using ES2002a video sequences described in Section 4.1.1.

Two Clear-MOT metrics were applied for each video sequence as well as for the overall video sequences—the multiple object tracking accuracy (MOTA) and the multiple object tracking precision (MOTP) [83]. Standard evaluation scripts which support Clear-MOT were used (https://github.com/cheind/py-motmetrics). Error calculations were made using the Euclidian distance between hypothesized (output from the method) and manually labelled person centres.

The correspondence threshold T strongly affects the behaviour of MOTA/MOTP measures. On one hand, setting T = *∞* indicates that all correspondence between hypotheses and ground truths are valid no matter how large the distance is. This in turn affects the accuracy of MOTA to measure the correct number of detections. Conversely, setting T approximately near to zero, all objects will be considered as missed; therefore, MOTA and MOTP become useless. In our experiments, we used T = 40 cm (an average person’s width in the data set).

##### Experimental Results and Discussion

Results for the MOTA and MOTP are shown in Table 2.

The multiple object tracking accuracy (MOTA) shows how many errors the tracker made in terms of false negative (FN), false positive (FP) and mismatches. The best performing sequences in terms of tracking accuracy, Seq01, Seq04 and Seq05 at 100%, 100%, 97.70% accuracy respectively. Seq01 and Seq05 accuracy show the robustness of our algorithm to detect and track meeting participants when they are sitting (i.e., making very small motion); note that it is considered to be quite difficult to detect a person when he/she is sitting [85]. Seq05’s FP and FN errors were the second best. FP and FN errors can be attributed to track confusion with another person (i.e., people who are very close or touching).The accuracy achieved in Seq04 demonstrates the strength of our method to detect and track people exhibiting varying motion (i.e., slow/sitting versus fast/walking).

The next highest accuracy score reported in Seq02 at 96.70 accuracy, which illustrated how well our method perform in some tracking challenge events such, people close to each other, touching, passing each other (partial overlap) and when their motion is different (sitting, moving, standing).

Seq03 and Seq06 reported the least tracking accuracy at 80% and 77.40% respectively. The biggest challenge for the tracking method (i.e., the full overlap between two people) occurred in Seq03 which cause high FN rates at 0.1 compared with other sequences. Tracking accuracy decreased in Seq06 due to the high FP rates at 0.16 as people start leaving and our method did not consider any track termination. In addition, a mismatch error was reported as a track of one person was lost and incorrectly reinitialized with a different identity.

The multiple object tracking precision (MOTP) is the total error in estimated position for associated ground truth-hypothesis over all frames divided by the total number of matches found. In terms of precision, the least precision error was in Seq04 and Seq03 at 15 pixels. It shows the precision of our algorithm in scenarios where there are different motions of people with less overlapping. While Seq02 and Seq06 reported the highest localization error at 20 and 22 pixels respectively which can be attributed to people close to each other which leads to overlapping challenges (partial overlap “one is sitting and other is moving” or full overlap “people walk close to each other”). It should be noted that the goal of our algorithm is robustness rather than precision, that is, we favour an approach which minimises FN/FP rather than maximising locational accuracy.

The MOTA as well as MOTP were calculated for the entire sequences as shown in Table 2. Our algorithm achieved a high accuracy score at 89.2% with low false negative and false positive rates at 0.051 and 0.056 respectively. In addition to a decent MOTP error was reported at 18 pixels. This demonstrates that our algorithm robustly estimates the trajectories of the meeting participants in the AMI video data from the overhead camera.

#### 4.1.4. Tracking Evaluation Results Using Track Quality Measures

##### Test Objective and Parameters

The same test objective and parameters described in Section 4.1.3 were applied.

##### Experimental Results and Discussion

Track quality measures evaluate how much of the object’s trajectory is recovered by the tracking algorithm. Table 3 shows the results for the three track quality measures—mostly tracked (MT), partially tracked (PT), and mostly lost (ML). An object is considered mostly tracked when it is found for at least 80% of its presence. An object is said to be mostly lost when it is tracked for less than 20% of its total length. All other tracks are partially tracked (PT).

As shown in Table 3, MT = 4 in Seq01, Seq02, Seq04, Seq05, and Seq06 which indicates that all four meeting participants were tracked successfully for at least 80% of its life span. In seq03, the MT = 3 and PT = 1. This shows that one person was partially tracked (this was as result of overlapping, where two people passed each other). The mostly lost trajectory ML = 0 in all sequences.

#### 4.1.5. Comparison with Published Results of Multiple People Tracking in Clear-Mot Workshops 2006

The testing results of all tracking algorithms developed at the UKA [20], AIT [29] and UPC [28] sites in the context of the CHIL project were evaluated in the Clear 2006 workshop using the interaction seminar database (meeting videos) recorded during the CHIL project. Two testing conditions were involved—firstly, without background images available which made foreground segmentation challenging. Secondly, background images were used (previously recorded background images of the empty meeting room) [31].

According to the results published in the Clear 2006 workshop [31], the best performing tracking system was UKA (top view) which was based on a single top camera view—this achieved 51.12% and 62.79% MOTA and precision at 217 mm and 201 mm for the two testing conditions. Identical comparison of these approaches to our method described in this paper is not possible as we did not get access to the CHIL data set. However, our approach achieves higher MOTA and precision compared with the best performing system UKA (top view) in the Clear 2006 evaluation test workshop for visual tracking methods. The evaluation was performed based on the AMI entire video sequences (Section 4.1.1). Our methods reached 89.2 MOTA and MOTP 121.68 mm.

#### 4.1.6. Comparison with Baseline and Top Performing Tracking Methods

##### Test Objective and Parameters

**Figure 8:**
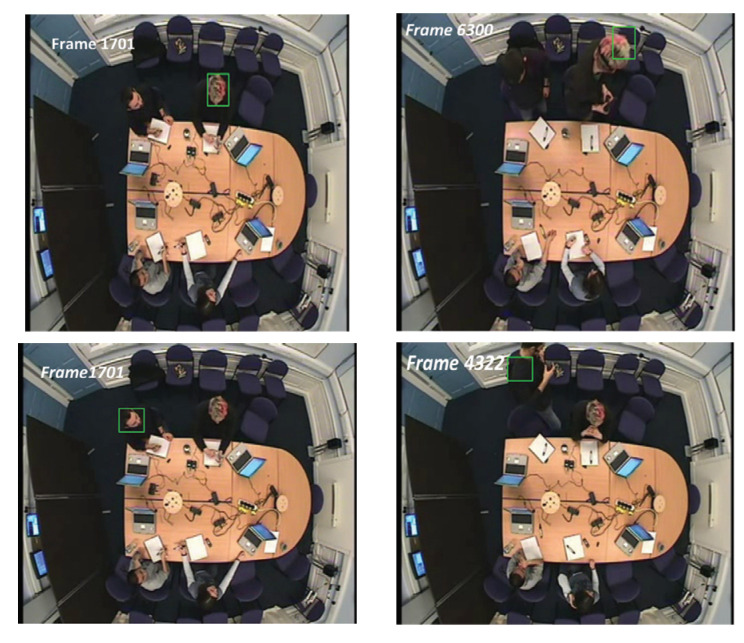
Example results produced by a generative tracker. The first row illustrates detecting the head of a person based on his head template. The detection results were very robust when the person started moving even though his head shape changed with respect to the viewing angle of the camera. The disadvantage of template matching is that strong colour features are needed to detect the head when its shape changes. The second row again shows good results where a meeting participant sits with only small changes to his head shape. However, the technique fails when the person starts moving, due to larger changes in the appearance and in the absence of strong colour features.

Using the AMI meeting video, we compare our algorithm, denoted by FD_Corner, with several baseline and state of the art tracking methods—online multiple instance learning (MIL) [64], Kernelized Correlation Filter (KCF) [63] and discriminative correlation filter with channel and spatial reliability (CSRDCF) [79].The discriminative trackers were chosen as our testing results showed that the generative tracker (e.g., template matching) fail in an environment when an object’s features are similar to the background and it needs a strong appearance model, see Figure 8. We perform quantitative and qualitative evaluations on the entire video sequence and separately on sequences exhibiting different tracking challenges as described in Section 4.1.1. We report the evaluation results by means of Clear-MOT, with accuracy and precision, described in Section 4.1.3. The results of the performance of the MIL, KCF and CSRDCF trackers were obtained through their implementation provided by an Open CV library with default settings.

##### Experimental Results and Discussion

- Quantitative Analysis on the Entire Video Sequence

Table 4 depicts the evaluation results of our developed tracking method FD_Corner and three other tracking methods (MIL, KCF, CSRDCF). Our approach, FD_Corner achieves the best performance when tracking people in the meeting video captured by an overhead camera at 89.2% accuracy (MOTA), with mismatch error equal to 1. The second highest accuracy was reported by CSRDCF at 86% accuracy.

CSRDCF considers only foreground pixels to update its correlation filter on each frame, in addition to Histogram of gradients (HOG) and colour-names as features. This in turn reduces the tracking errors compared with KCF and MIL. The MIL tracker uses Haar-like features [86].

In terms of precision error, the FD_Corner tracker achieves the lowest localisation error, at 18.67, followed by the KCF tracker, at 18.87 pixels. The KCF reports a good localisation performance, as it used a circulant matrix which allows it to consider samples from all locations in filter training [77].

Overall, the highest accuracy obtained by our tracker shows the effectiveness of the developed tracking-by-detection framework. It performs tracking-by-detection with no need for the training (learning) step involved in most of the discriminative trackers (e.g., MIL, KCF, CSRDCF). The FD_Corner tracker relies on Adaptive Accumulated Frame differencing (AAFD) with appearance model (corner features) in every time step in order to localize the object at each step with no prior knowledge. The updating step for the other discriminative trackers is based on a correlation filter and may fail to locate the object when there is major change in the object’s appearance (e.g., its features are not clear in a cluttered background). Our tracker robustly finds and tracks people when there are changes in their appearance, using a combination of corner features with motion data (AAFD).

- Quantitative Analysis on Each Video Sequence

Quantitative comparisons of our tracker with the three other trackers in each video sequence are shown in Table 5 with different track challenges, to demonstrate the robustness of each tracker. All trackers reported high accuracy in seq01 and seq05. In these sequences, meeting participants are sitting and there is no major change in their appearance.

In seq02, seq04, seq06, meeting participants are moving in a meeting room (e.g., going to the whiteboard, leaving the meeting room). These sequences involve changes in the visual size of people as well as appearance change challenges. Our tracker achieves the best accuracy at 96.70%, 100% and 77.40% in seq02, seq04, seq06 respectively compared with other discriminative trackers where their performance decreases with changes in the target’s appearance in a cluttered background. In seq03 where full overlap occurs between two meeting participants, the MIL tracker and FD_Corner tracker report the highest accuracy at 80%.

- Qualitative Evaluation

**Figure 9:**
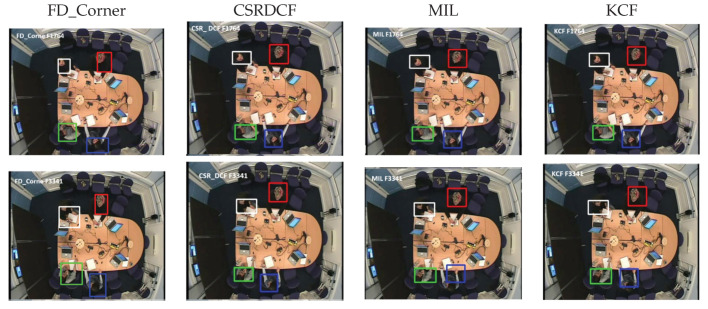
Qualitative evaluation of our approach FD_Corner in comparison with three baseline and state of art trackers. Tracking results for two examples from the meeting video sequence 01 shows that all trackers exhibit relatively good performance in a sequence whith no major appearance change.

**Figure 10:**
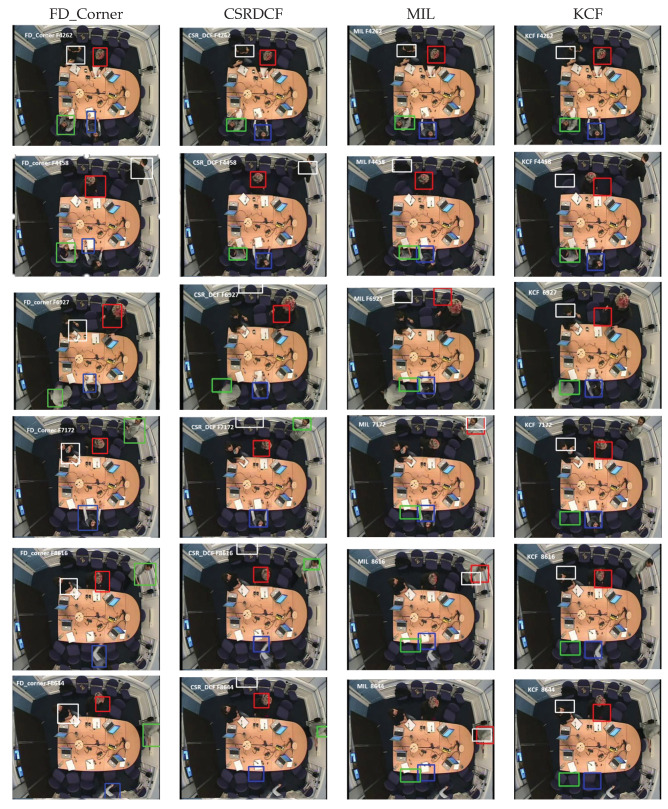
Qualitative evaluation of our approach FD_Corner in comparison with three baseline and state of art trackers on sequence 02 in terms of appearance change. Our tracker FD corner outperforms discriminative correlation filter with channel and spatial reliability (CSRDCF), Kernelized Correlation Filter (KCF), multiple instance learning (MIL) trackers in scenarios where people start moving in the meeting room and their appearance change significantly due to partial occlusion (person’s head out of camera view).

This section presents the qualitative evaluation of our tracking method FD_Corner in comparison with other tracking methods. Figure 9 and Figure 10 show examples of tracking with FD_Corner, CSRDCF, MIL and KCF based on seq02 (people start moving in the meeting room, therefore their appearance and scale is changed) and seq01 (people are sitting around the table so there is no significant appearance change challenges).

Figure 10 presents visual examples of all four trackers on seq02 where people are moving in the meeting room and their appearance changes significantly. The first example in Figure 10 (F4262) shows tracking at the beginning frame when a person (white rectangle) starts moving to whiteboard.

The tracking bounding boxes of the MIL and KCF tracker suffer drifting as shown in the second example; second row in Figure 10 (F4458). Here the target is turning his back to the camera view and his head is partially outside the camera’s view. FD_Corner and CSRDCF track the person correctly in this frame.

The third example, F6927 shows the CSRDCF tracker fails when the person returns to his seat (due to foreground colour features similar to the background).The tracking result on frame F6927, F7172 shows that, the second person (Red rectangle) and the third one (Green rectangle) are robustly tracked by FD_Corner and CSRDCF when they are moving to whiteboard and coming back to their seat while MIL and KCF tracker fails. The KCF tracker for the second person recovers (Red rectangle) when he is sitting—see F7172.

The last two examples, F8612 and F8644 demonstrate the robustness of our tracker FD_Corner to track all meeting participants. The bounding rectangle of the CSRDCF, MIL and KCF tracker drift for the fourth person (Blue rectangle).

Tracking results show that FD_Corner achieves relatively robust performance in a sequence where target appearance changes significantly or is partially out of view (e.g., participant’s head out of camera view) compared with other trackers. It can be seen that, the bounding box of KCF, MIL, CSRDCF trackers drift, which results in tracking failure.

- Robustness to Initialisation

One of the challenges for visual tracking algorithms is robustness to initialisation (i.e., a tracker is considered robust to initialisation if its performance is not affected when the tracker is initialised at different starting frames). The initial bounding box for a tracker is sampled temporally (at different starting frames) to evaluate the robustness of the tracker [73]. To evaluate the initialisation robustness, two experiments were performed. Firstly, evaluation was performed by using different video sequences, where a tracker is initialized on the first frame of a sequence (therefore the tracker bounding box is sampled temporally different as the starting frame on each sequence is different) and MOTA accuracy is reported, as described in Section 4.1.6. Furthermore, a tracker’s robustness to initialisation was evaluated by averaging the tracker MOTA accuracy of all sequences. As shown in Table 6, our tracker FD_Corner was the most robust tracker to initialisation followed by CSRDCF at 94.36 and 86.40 accuracy.

The next experiment was performed using seq02 which presented appearance change and visual size variation plus background clutter. Trackers were evaluated by initializing at a different frame on the sequence 02 and run to the end of the sequence (i.e., one segment). Then, the average multiple object tracking accuracy (MOTA) of all segments was calculated to assess the tracker robustness to initialisation—see Table 7.

To sum up, in both evaluations, FD_Corner was the most robust to initialisation. This is due to the fact that we perform detection in every frame while requiring no prior knowledge of object features. In the discriminative trackers MIL, KCF, CSRDCF, in the first frame, the filter/classifier is initialized by learning with an image patch obtained based on the initial position of the object. In some environments (e.g., meeting video captured from the overhead camera), object features are not clear and have similar colour to the background which leads to inaccurate initialisation. In the next frame, the filtering image is tested with an input image to find the object location and the filter is updated based on the object’s new location. This may cause tracker drift when there is major change in the object’s appearance in a cluttered background environment which eventually leads to tracker failure. Finally, we notice that, increasing the size of the bounding box during tracker initialisation leads to a performance decrease for all discriminative trackers as many background pixels are included in the initial representation. This indicates that these trackers are more sensitive to background clutter.

### 4.2. Attribute-Based Evaluation on Generic Visual Object Tracking Dataset

Evaluating a tracking algorithm itself is a challenge. Many factors can affect the performance of a visual tracking algorithm (e.g., scale variation, camera motion, background clutter, and occlusion). In a recent visual object tracking benchmark, the Online Tracking Benchmark (OTB), sequences are annotated with different challenges such as illumination variation, occlusion, camera motion and size change [73,74]. This describes the tracking challenges a tracker will face and allows better performance analysis of the weaknesses and strengths of a tracker based on these attributes.

#### 4.2.1. Test Objective and Parameters

We perform our attribute-based evaluation on 6 selected indoor/outdoor video sequences from the OTB. All video sequences and ground truths are publicly available on the Online tracking benchmark website (http://cvlab.hanyang.ac.kr/tracker_benchmark//datasets.html) [73]. The video sequences are annotated by different tracking attributes (i.e., Scale variations-SV, Deformation-DEF, Fast motion-FM, out-of-plane rotation-OPR, Background clutter-BC, Illumination variation-IV, Occlusion-OCC, in-plane rotation-IPR, Low resolution-LR)—see Table 8 for more information.

We construct subset sequences with different dominant attributes to evaluate the performance based on these challenging attributes. Five experiments are performed on subsets with background clutter (BC), deformation (DEF), illumination variation (IV), scale variation (SV) and occlusion (OCC) dominant attributes to evaluate the performance of the tracker for each challenging factor.

Our FD_Corner tracker is compared with the top performing tracker for each of the BC, DEF, IV, SV and occlusion attributes. In terms of precision plot, according to the online tracking benchmark [73], one of the top performing trackers for DEF, IV, SV, OCC attributes was Tracking-learning-detection- TLD [66,75]. Our tracker is also compared with KCF tracker based on color features which achieved the best performing tracker on BC, DEF, IV and OCC attributes based on the OTB dataset [63]. In addition to the comparison to a Generic Object Tracking Using Regression Networks(GOTURN) as it is one of the key trackers which proposed an alternative deep learning approach. GOTURN uses the deep regression network to learn the generic relationship between the object appearance and the object location by estimating the motions [87]. The results of the performance of the TLD, KCF and GOTURN trackers were obtained through their implementation provided by an Open CV library with default settings.

#### 4.2.2. Experimental Results and Discussion

Performance is evaluated using precision plots, which show the frame ratio, that is, the distance between estimated object location and the ground truth within a given threshold. To summarize these plots, we consider precision (frame ratio of corrected matches) at threshold=20 to rank the tracker.

**Figure 11:**
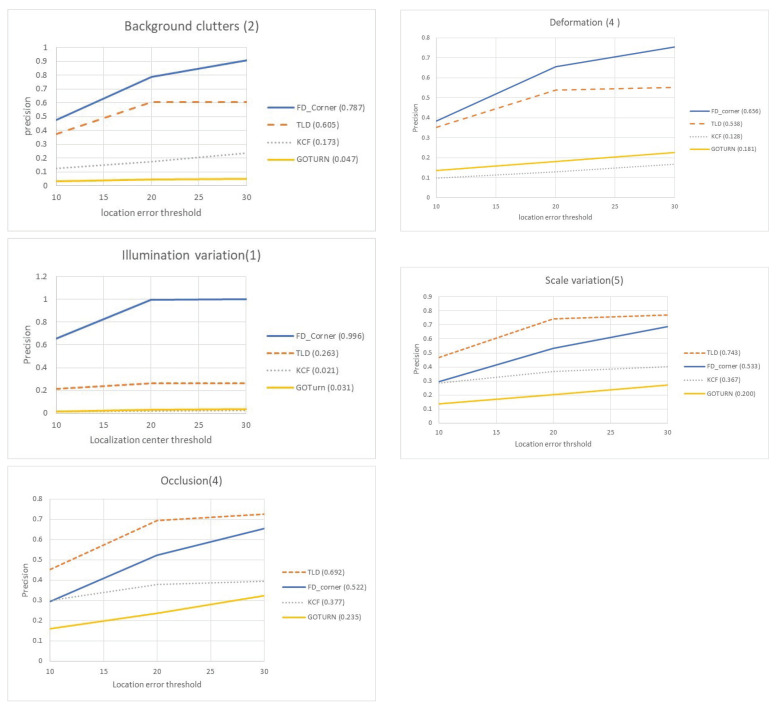
The precision plot for sequences with attributes: background clutter, deformation, illumination variation, scale variation and occlusion. Our FD Corner tracker perform favourably on background clutters, deformation and illumination variation attributes.

Figure 11 shows the precision plot of different attributes over a range of thresholds. Our FD_Corner tracker scored the best on the three attributes—background clutter, deformation and illumination variation at 0.78, 0.65 and 0.99 precision, outperforming by 18%, 12%, and 73% the second best tracker (the TLD). The robustness of FD_Corner to background clutter, deformation and illumination variation tracking challenges is explained by our use of motion features which robustly detect objects when their appearance features are not clear or change significantly. Performance degradation of the TLD can be related to sampling of negative samples when the target has a similar appearance to the background. The performance is also decreased when the sequence contains objects similar to the target.

The experiments on sequences with scale variation and occlusion show that the TLD performs the best, followed by FD_Corner. This is can be attributed to the TLD re-detection module. The current version of our tracker may not perform robustly in the case of occlusion. The FD_Corner can robustly track people when their appearance changes (e.g., scale variation) as discussed in Section 4.1.6. However, the FD_Corner reports a precision of 0.53 in the subset with scale variation—this is largely due to the fact that most of the videos used in the scale variation attribute test also have occlusion challenges.

The lowest performance is reported by the KCF and the GOTRUN trackers. The performance of the GOTURN heavily depends on the appearance model of the tracking target and it can be improved by including samples of people captured from a top view camera.

## 5. Conclusions

In this paper, a tracking-by-detection approach to track meeting participants from an overhead camera is introduced. In this, a novel adaptive accumulated frame differencing (AAFD) approach is applied to find the region of movements for each person over varying temporal scales, where a large temporal window size is used to find slow moving objects and a small temporal window size is used to detect fast moving objects. Corner features are introduced as a different form of evidence (feature based rather than motion based) in order to track meeting participants robustly. A score is calculated for each extracted corner, indicating those corners which coincide with movement; the corners with high scores are returned and the minimum area rectangle is then found for a region of pixels.

To the best of our knowledge, this is the first work for people tracking in meeting contexts to have used the AMI meeting video captured using an overhead camera. We present and discuss testing analysis of our developed method in a meeting context using multiple object tracking metrics, and per-attribute analysis using a general (non-meeting) data set, that is, the online tracking benchmark (OTB). Quantitative and qualitative results of our FD_Corner tracker compared with a number of state the art algorithms show that our tracker exhibits promising robustness when tracking meeting participants from the overhead camera when there is no prior background model available. Furthermore, quantitative results on the general purpose datasets indicates that our tracker performs better than state-of-the-art trackers under three specific challenges—background clutter, deformation and illumination variation.

## 6. Limitations and Future Work

The current version of our tracker does not perform well in the case of full overlap (i.e, where one person walks past another). One promising line of enquiry that we are working on is to use the vector dot product of (i) a person’s normalised tracked velocity and (ii) the normalised velocity indicated by optical flow. This allows us to reject extracted corner points if the direction of movement suggested by optical flow disagrees with the known direction of movement of the person over a number of previous frames.Furthermore, Kalman filtering can be used when blob overlapping is detected to address the overlapping challenge.

The result of the tracker developed in this paper can be used to construct the context information about the scene (i.e., the number, location and movements of participants) as a timeline. This timeline can be used for example, in an action recognition application to control information obtained from personal cameras (i.e., discarding frames when someone is moving behind the owner of a personal camera), in addition to exploiting this high-level information to generate a meeting browser.

The motion model, which relates the location of the object over all frames in a sequence, and appearance model, which evaluates the presence of the object at a specific location, are the most important components for any tracking algorithm. While motion modelling may be useful for tracking, most tracking methods omit this component. Good estimation of object location can reduce search region for the tracker and improve tracking robustness [74]. Our FD_Corner approach could be employed as a robust motion model component, in order to improve traditional feature based (appearance) algorithms by assisting their blunder recovery.

The combination of AAFD with corner features presented in this paper may also be used as features to train a traditional classification method such as Support Vector Machines (SVMs).

The combination of motion data with corner features may also become a primary target for machine learning applications into the future. Generally, a feature descriptor for video analysis can be categorised into handcrafted feature descriptors and deep learning descriptors. The handcrafted descriptors extract the features based on the manually designed algorithms, while the deep learning descriptor learns features automatically from a video.

Recently, Convolutional Neural Networks (CNNs) have achieved great success in the fields of object detection in computer vision. CNNs are essentially used to obtain spatial features from the static image. A single frame-based CNN is capable of extracting spatial information from the video sequence, which is very important to extract the appearance features. However, motion information is not considered [88]. Furthermore, a two-stream CNN architecture is proposed in Reference [89] which combines spatial- and optical-based motion information. However, this architecture uses optical flow, which does not perform adequately when objects are moving slowly. The combination of deep spatial features using CNN and handcrafted spatiotemporal features implemented in this paper (a combination of motion data with corner features) could be applied for object detection. This could be achieved by concatenating both the CNN output and the handcrafted features, then feeding this into a classifier (i.e., SVM). 

## Figures and Tables

**Table 1:** Challenges in the ES2002a video. In these 6 sequences, we identify the presence (Y) or absence (N) of specific events.

	Seq01	Seq02	Seq03	Seq04	Seq05	Seq06
Total No of frames	2484	4533	104	2775	17875	1775
event: sit down	Y	Y	Y	Y	Y	Y
Event: occlusion (passing each other)	N	Y	N	N	N	Y
Event: occlusion (walk past each other)	N	N	Y	N	N	Y
Event: leaving	N	N	N	N	N	Y
Event: touching /people close to each other	Y	Y	Y	N	Y	Y
Event: stand up(walking)	N	Y	Y	Y	N	Y
Description	All people are sitting	People are sitting and start moving to the whiteboard. Occlusion( passing each other)	occlusion between two people (Fully overlapping)	People are sitting, while one is moving with no overlapping	All people are sitting	Some participants leaving the meeting room (walking near each other when they are leaving)

**Table 2:** Results for the Clear-multiple object tracking (MOT) evaluation measurement.

Sequence #	MOTP	FN	FP	Mismatches	MOTA
Sequence 01	17	0	0	0	100%
Sequence 02	20	12	12	0	96.70%
Sequence 03	15	2	2	0	80%
Sequence 04	15	0	0	0	100%
Sequence 05	18	33	33	0	97.70%
Sequence 06	22	16	42	1	77.40%
Overall	18	241	267	1	89.20%

**Table 3:** Results for track quality measures Mostly track (MT), partially tracked (PT), and mostly lost (ML).

Sequence #	MT	PT	ML
Sequence 01	4	0	0
Sequence 02	4	0	0
Sequence 03	3	1	0
Sequence 04	4	0	0
Sequence 05	4	0	0
Sequence 06	4	0	0
Overall	4	0	0

**Table 4:** Comparison of the baseline and stare of art trackers with frame difference (FD) Corner: Clear-MOT evaluation measurement on the entire video sequence.

Tracking Method	Clear-MOT Metrics				
	MOTA	FN	FP	Mismatches	MOTA
**FD_Corner**	18.67	241	267	1	***89.2%***
**MIL**	35.69	902	925	7	61.00%
**KCF**	18.87	329	357	5	85.30%
**CSRDCF**	22.47	317	340	3	86.00%

**Table 5:** Comparison of the baseline and stare of art trackers with FD corner: Clear-MOT evaluation measurement (Multiple object tracking precision (MOTP) and multiple object tracking accuracy (MOTA) on each video sequence.

Sequences/ Clear-MOT Metrics	Tracker							
	Our FD_Corner		KCF		MIL		CSRDCF	
	MOTP	MOTA	MOTP	MOTA	MOTP	MOTA	MOTP	MOTA
**Seq01**	17	100.00%	6	100.00%	11	100.00%	11	100.00%
**Seq02**	20	96.70%	16	55.20%	25	65.50%	18	72.10%
**Seq03**	15	80.00%	20	70.00%	12	80.00%	12	50.00%
**Seq04**	15	100.00%	8	49.30%	24	73.90%	17	100.00%
**Seq05**	18	97.70%	20	100.00%	27	98.70%	19	97.80%
**Seq06**	22	77.40%	25	55.60%	23	44.10%	18	62.10%

**Table 6:** Comparison of the baseline and stare of art trackers with FD corner in terms of robustness to initialisation. Our tracker FD_Corner was the most robust tracker to initialisation.

Sequences/MOTA	FD_Corner	KCF	MIL	CSRDCF
**Seq01**	100.00%	100.00%	100.00%	100.00%
**Seq02**	96.70%	55.20%	65.50%	72.10%
**Seq04**	100.00%	49.30%	73.90%	100.00%
**Seq05**	97.70%	100.00%	98.70%	97.80%
**Seq06**	77.40%	55.60%	44.10%	62.10%
**Average**	94.36	72.02	76.44	86.40

**Table 7:** Comparison of the baseline and stare of art trackers with FD_Corner in terms of robustness to initialisation. A tracker was initialized at different frames on sequence 2. FD_Corner tracker obtain the best result.

Segments #	FD_Corner	KCF	MIL	CSR-DCF
**Segm01 F4150-F8650**	96.70%	55.20%	65.50%	72.10%
**Segm02 F5000-F8650**	99.30%	58.50%	57.30%	70.10%
**Segm03 F6300-F8650**	98.90%	52.60%	65.80%	63.20%
**Average**	98.30%	55.43%	62.87%	68.47%

**Table 8:** Example of Online tracking benchmark video sequences.

Sequence	Total NO of frames	Attributes
**1. Crossing**	95	SV, DEF, FM, OPR, BC
**2. Crowds**	322	IV, DEF, BC
**3. Human5**	688	SV, OCC, DEF
**4. RedTeam**	1893	SV, OCC, IPR, OPR, LR
**5. Walking**	387	SV, OCC, DEF
**6. Walking2**	475	SV, OCC, LR

## Abbreviations

The following abbreviations are used in this manuscript:

ROI	Region of movement
FD_Corner	Frame differencing Corner
ROI	region of interest
AAFD	Adaptive Accumulated Frame Differencing
MIL	online multiple instance learning
KCF	Kernelized Correlation Filter
CSRDCF	discriminative correlation filter with channel and spatial reliability
TLD	Tracking-learning-detection
